# Gene Polymorphisms of *ADIPOQ* +45T>G, *UCP2* -866G>A, and *FABP2* Ala54Thr on the Risk of Colorectal Cancer: A Matched Case-Control Study

**DOI:** 10.1371/journal.pone.0067275

**Published:** 2013-06-27

**Authors:** Xiaoqin Hu, Ping Yuan, Jin Yan, Fei Feng, Xiaoling Li, Wenhui Liu, Yanfang Yang

**Affiliations:** 1 Department of Epidemiology, West China School of Public Health, Sichuan University, Chengdu, Sichuan, China; 2 Department of Intestinal Surgery, Sichuan Cancer Hospital, Chengdu, Sichuan, China; MOE Key Laboratory of Environment and Health, School of Public Health, Tongji Medical College, Huazhong University of Science and Technology, China

## Abstract

As insulin resistance (IR) is an established risk factor for colorectal cancer (CRC), we explored the association between each of the IR-related gene polymorphisms of adiponectin (*ADIPOQ*) rs2241766, uncoupling protein 2 (*UCP2*) rs659366, and fatty acid-binding protein (*FABP2*) rs1799883 and CRC risk. Genotyping of blood samples and collection of lifestyle and dietary habits were performed for 400 case-control pairs. Unconditional logistic regression (ULR) was applied to assess the effects of the three single nucleotide polymorphisms (SNP), environmental factors. Both ULR and generalized multifactor dimensionality reduction (GMDR) were used to test the gene-gene and gene-environment interactions on CRC risk. Subjects carrying the *ADIPOQ* rs2241766 TG+GG genotype had a higher CRC risk than those carrying the TT genotype (OR = 1.429, 95% CI 1.069–1.909). The additive and multiplicative interactions between *ADIPOQ* rs2241766 and *FABP2* rs1799883 on CRC were found by ULR (RERI = 0.764, 95%CI 0.218∼1.311, AP = 0.514, 95%CI 0.165∼0.864, S = −1.745, 95%CI is unachievable, and P_multi_ = 0.017, respectively). Furthermore, the high order gene-gene interaction of the three SNPs were found by GMDR (P = 0.0107). A significant dosage effect with an increasing number of risk genotypes was observed as the risk of CRC increased (P_trend_ = 0.037). In GMDR, the gene-environment interaction among the three SNPs and red meat consumption on CRC risk was significant (P = 0.0107). Compared with subjects with low red meat consumption and null risk genotypes, those with high-red meat consumption and three risk genotypes had 3.439-fold CRC risk (95% CI 1.410–8.385). In conclusion, the results showed that the *ADIPOQ* rs2241766 TG+GG genotype increased CRC risk. Given the complexity of the carcinogen for CRC, *ADIPOQ* rs2241766, *UCP2* rs659366, *FABP2* rs1799883 and red meat consumption potentially worked together in affecting CRC risk.

## Introduction

Colorectal cancer (CRC) is the second most commonly diagnosed cancer and the fourth leading cause of cancer deaths worldwide [1]. Considering the westernization of dietary habits and lifestyle changes, the incident rates of CRC in Eastern Asia and in Eastern Europe, which historically had the lowest rates, have remarkably increased in recent years [2]. In China, the incident rates of CRC grew from 15.0/10^5^ to 32.5/10^5^ among men and from 9.7/10^5^ to 26.7/10^5^ among women, within the period of 2005 to 2007, whereas mortality rates increased from 8.6/10^5^ to 15.6/10^5^ among men and from 5.4/10^5^ to 12.7/10^5^ among women [3].

Although the mechanism of CRC remains unclear, many researchers have suggested that environmental and genetic factors work together on the proliferation of CRC [4]. Western dietary habits, such as high-red meat consumption [5], and a sedentary lifestyle have been proven to play important roles in the development of CRC [6]. Unlike health people, individuals suffering from obesity and type 2 diabetes mellitus (T2DM) have high incidence and mortality rates of cancer [7], [8], whereas both obesity and T2DM are associated with insulin resistance (IR) [9], [10]. In the 1990s, McKeown-Eyssen [11] and Giovannucci [12] originally proposed a hypothesis of “insulin resistance-colon cancer”.

IR is characterized by compensatory hyperinsulinemia resulting from impaired biological response to insulin action [13] and is believed to increase the risk of hyperlipidemia [14], T2DM [10], and cancer [15]. Although the mechanisms for the association between IR and cancer risk are unknown, 3 potential mechanisms explain the relationship. First, hyperinsulinemia and a high insulin-like growth factor-1 (IGF-1) level, which are caused by increased insulin, promote cell proliferation and inhibit apoptosis, contributing to the suppression of the hepatic synthesis of the sex hormone-binding globulin [16], [17]. Second, elevated reactive oxygen species frequently associated with IR can damage DNA by mutagenesis and carcinogenesis [18]. Third, the inflammatory environment of patients with obesity and T2DM can promote carcinogenesis [19]. The mechanisms mentioned above are also appropriate for the association between IR and CRC risk, and many studies elaborate on the relationship. A study on gastrin gene knockout (GAS-KO) mice [20] indicated that the loss of amidated gastrins may increase hyperinsulinemia and colon carcinogenesis and, furthermore, colon carcinogenesis, as a result of hyperinsulinemia. Limburg et al. [21] conducted a prospective study among male smokers, suggesting that hyerinsulinemia was a CRC risk factor. A recent study performed by Ortiz et al. [22] formulated a conclusion that central obesity and IR played important roles in the early stage of colorectal neoplasia, particularly among men. Komninou et al. [23] performed a review on the contribution of IR to colon carcinogenesis and provided strong support for the hypothesis.

IR is a complex phenotype with strong genetic predisposition. A number of genes are related to IR, mainly including the adiponectin gene (*ADIPOQ*), uncoupling protein 2 (*UCP2*), fatty acid-binding protein 2 (*FABP2*), β_3_ adrenergic receptor (*β_3_-AR*), calpain 10 gene (*CAPN-10*), and insulin receptor (*INSR*). We assumed that an SNP, which sustained its association with IR in several studies, might be a good candidate to explore the genetic role of the selected genes on CRC risk in the current study. After doing extensive article review, we chose 3 of the most studied hotspot genes and their respective loci, the impacts of which on IR were putative, to investigate the association between gene polymorphisms related to IR and the susceptibility to CRC. These single nucleotide polymorphisms (SNP) are *ADIPOQ* +45T>G (rs2241766), *UCP2* -866G>A (rs659366), and *FABP2* Ala54Thr (rs1799883). Until now, except for *ADIPOQ* rs2241766, no research has been conducted on the relationships between each of *UCP2* rs659366 and *FABP2* rs1799883 and CRC risk.

To our knowledge, this is the first study reporting the association of gene polymorphisms of the IR pathway with CRC risk. High red meat consumption has been demonstrated to increase the risk of IR [24] and CRC [5]. The gene-gene and gene-environment interaction is a hot topic in genetic epidemiology [25–27], thus, we explored the association of IR-related gene polymorphisms and CRC risk to identify susceptible genes and observe the gene-gene and gene-red meat consumption interaction on CRC.

## Materials and Methods

### Study Population

Eight hundred (400 cases with CRC and 400 controls) subjects were enrolled. All of them were Han Chinese, aged 20 years to 80 years old, and had been living in Sichuan for at least 20 years. Cases with newly histopathologically diagnosed primary CRC between July 2010 and May 2012 were recruited from the Sichuan Cancer Hospital (Chengdu, Sichuan, China). The cases include 268 (67.0%) subjects with rectal cancer and 132 (33.0%) subjects with colon cancer. Controls were selected from healthy people receiving routine medical examinations at the Zhonghe Community Health Service Center (Chengdu, Sichuan, China) during the same period as the cases. The cases and the controls did not have any earlier history of cancer. Controls were matched 1∶1 to cases by gender and age (±3 years) upon enrollment.

Written informed consent for an interview and 2 ml peripheral whole blood were obtained from each study participant. The study protocol was approved by the Institutional Review Board of Sichuan University.

### Exposure to Environmental Factors

Well-trained interviewers used a structured and validated questionnaire to collect information from subjects personally and followed a written protocol to ascertain and reduce monitoring, interviewer, and recall bias. The questionnaire inquired on demographic features and potential CRC risk factors that include family history of CRC, specifically among first and second degree relatives, sedentary lifestyle measured on the number of sitting hours per day, smoking, alcohol-drinking, and tea-drinking habits measured by duration, type, and consumption, and red meat consumption, which include beef, lamb, and pork, measured by frequencies per week. Smoking, alcohol-drinking, and tea-drinking habits were defined as smoking more than one cigarette per day for at least 6 months, drinking alcohol more than two times per week for at least 6 months, and drinking tea more than one times per day for at least 6 months, respectively. Red meat consumption was defined as more than 50 g at one time according to the dietary habits of Sichuan residents and measured by frequencies per week. The medians of red meat consumption for the cases and controls were 9 times/week and 7 times/week, respectively. According to the median in controls and the advised 50–75 g/day red meat consumption by Dietary Guideline and Balance Diet Pagoda for Chinese Residents, we divided red meat consumption into dichotomized variables with 7 times/week as the cut off value. Questions on the lifestyle and dietary habits were asked by general condition in reference to the 10 years before disease diagnosis.

### DNA Extraction and Genotyping

The cases and controls were requested to provide 2 ml peripheral whole blood, which was collected in trisodium citrate and stored at −40°C. Genomic DNA was extracted from whole blood using SE Blood DNA Kit (Omega Bio-Tek, Guangzhou, China), according to the manufacturer’s instructions. As described previously [28], [29], [30], the polymerase chain reaction-restriction fragment length polymorphism (PCR-RFLP) method was applied to detect the *ADIPOQ* rs2241766, *UCP2* rs659366, and *FABP2* rs1799883 genotypes. The main parameters for the PCR-PFLP of the three SNPs are shown in Table 1.

**Table 1:** Primer sequences and reaction conditions for PCR-PFLP of *ADIPOQ* rs2241766, *UCP2* rs659366, and *FABP2* rs1799883.

Gene	Primer sequence	T^a^ (°C)	Enzyme^b^	Size^c^ (bp)
rs2241766	F^d^:5′-GAAGTAGACTCTGCTGAGATGG-3′	58	*Sma I*	T: 372
	R^e^:5′-TATCAGTGTAGGAGGTCTGTGATG-3′			G: 219, 153
rs659366	F:5′-CACGCTGCTTCTGCCAGGAC-3′	68	*Mlu I*	G: 295, 68
	R:5′-AGGCGTCAGGAGATGGACCG-3			A: 363
rs1799883	F:5′- ACAGGTGTTAATATAGTGAAAAG-3′	55	*Hha I*	Ala: 99, 81
	R:5′- TACCCTGAGTTCAGTTCCGTC-3′			Thr: 180

a Annealing temperature.

b Restriction enzyme.

c Enzyme-digested size.

d Forward primer.

e Reverse primer.

One hundred and sixty random samples (20% of the total subjects) were sequenced using an ABI 3730XL sequencer (Applied Biosystems, Invitrogen Trading Co., Ltd., Shanghai, China) to confirm the accuracy of genotype, and the concordance rate was 100%.

### Statistical Analysis

Unconditional logistic regression (ULR) was performed to estimate the effects of environmental factors on CRC. The genotype distributions of *ADIPOQ* rs2241766, *UCP2* rs659366, and *FABP2* rs1799883 were examined using the *χ^2^* goodness-of-fit test to verify the Hardy–Weinberg equilibrium (HWE) in the control group using the online software, SHEesis (http://analysis.Bio-x.cn/myAnalysis.php). The genotype variable was first analyzed as a categorical variable and then reanalyzed as a dichotomized variable by combining the heterozygous and homozygous genotypes of the variants, with the wild genotype as reference. The odds ratios (OR) with 95% confidence intervals (CI) for the three SNPs on CRC risk were calculated using ULR and adjusted according to family per capita annual income, family history of CRC, sitting hours per day, body mass index (BMI), and smoking, alcohol-drinking, and tea-drinking habits. For *ADIPOQ* rs2241766, *UCP2* rs659366, and *FABP2* rs1799883 with their respective genotype frequency, we calculated the statistical power for our sample size to detect an OR of 1.5 is as follows: 81.3%, 76.7%, and 77.6%, respectively.

One order interactions of gene-gene and gene-environment were detected by ULR with above mentioned variables as adjusted factors. All the three SNPs and red meat were divided into dichotomized variables by the method mentioned above, and the group with high CRC risk was represented by 1 and the other one was represented by 0. By crossing two dichotomized variables, a dummy variable was obtained for four categories: two for the presence of each factor alone (OR_10_ or OR_01_), one for the presence of both factors (OR_11_), and one for the absence of both factors (OR_00_) which was used as the reference in the regression model. The OR for multiplicative interaction was calculated by OR_multi_ = OR_11_/OR_10_×OR_01_. The P value for multiplicative interaction was calculated by comparing a full model including a multiplicative interaction term to a reduced model without an interaction term, using a likelihood ratio test. To estimate additive interaction, relative excess risk of interaction (RERI = OR_11_−(OR_10_+OR_01_−1)), attributable proportion of interaction (AP = RERI/OR_11_), and the synergy index (S = (OR_11_−1)/[(OR_01_−1)+(OR_10_−1)]) and their 95% CIs were calculated by bootstrapping which were detailed by Andersson et al. [31].

Generalized multifactor dimensionality reduction (GMDR, version 0.7, obtained from http://www.healthsystem. *virginia.edu/internet/addiction-genomics/software/*) was applied to analyze high order interaction of gene-gene and gene-environment. A number of parameters, including prediction error, sign test P value, and cross-validation (CV) consistency, were obtained. The model with the minimum prediction error, the maximum CV consistency score, and 0.05 or lower P value derived from the sign test was considered as the best model. Confounding factors including family per capita annual income, family history of CRC, sitting hours per day, BMI, smoking habit, alcohol-drinking habit and tea-drinking habit were included as covariates in the GMDR models.

Generally, logistic regression (LR) is considered having higher accuracy in analyzing one order interaction, while GMDR is more suitable to find the high order interaction [32]. So the one order interaction was recommended to follow the results of LR. The MDR is a non-parametric and genetic model-free approach for overcoming some of the limitations of LR (i.e. sample size limitation) to detect and characterize the gene–gene and gene–environment interactions [27], [33].

The Microsoft Access program was used for data entry and management, and SPSS18.0 (SPSS Inc., Chicago, IL) and GMDR v0.7 were used for statistical analysis.

## Results

### Characteristics of Participants

Among the participants, the minimum and maximum ages were 22.0 and 80.0 years, respectively. The mean ages were (55.73±11.08) years for the cases and (55.74±11.19) years for the controls (t = 0.010, P = 0.992). For the cases and controls, 233 (58.2%) were males and 167 (41.8%) were females. ULR indicated that having a family history of CRC and longer sitting hours per day (≥8 hours/day) were associated with increased CRC risk (OR = 3.808, 95% CI 1.775–8.171 and OR = 1.810, 95% CI 1.250–2.620, respectively), whereas habitual tea drinking decreased CRC risk (OR = 0.617, 95% CI 0.451–0.844). Compared with participants who had low-red meat consumption (≤7 times/week), a 1.870-fold (95% CI 1.392–2.512) increase of CRC risk was observed among those who had high-red meat consumption (>7 times/week). No association was identified between each of family per capita annual income, BMI, smoking habit, alcohol-drinking habit and CRC risk (Table 2).

**Table 2:** ORs and 95%CIs of main risk factors for CRC.

Variable	Cases N(%)	Controls N(%)	OR (95%CI)	P
Family per capita annual income (yuan/year)				
<10000	134(33.5)	118(29.5)	Reference	0.211
≥10000	266(66.5)	282(70.5)	0.819 (0.598–1.120)	
Family history of CRC				
No	365(91.2)	391(97.8)	Reference	0.001
Yes	35(8.8)	9(2.2)	3.808 (1.775–8.171)	
Sitting hours per day				
<8	296(74.0)	337(84.2)	Reference	0.002
≥8	104(26.0)	63(15.8)	1.810 (1.250–2.620)	
BMI				
<24	245(61.2)	231(57.8)	Reference	0.372
≥24	155(38.8)	169(42.2)	0.874 (0.651–1.174)	
Smoking habit				
No	226(56.5)	230(57.5)	Reference	0.485
Yes	174(43.5)	170(42.5)	1.135 (0.795–1.622)	
Alcohol-drinking habit				
No	262(65.5)	259(64.8)	Reference	0.564
Yes	138(34.5)	141(35.2)	0.899 (0.626–1.291)	
Tea-drinking habit				
No	228(57.0)	190(47.5)	Reference	0.003
Yes	172(43.0)	210(52.5)	0.617 (0.451–0.844)	
Red meat(times/week)				
≤7	144(36.0)	214(53.5)	Reference	<0.001
>7	256(64.0)	186(46.5)	1.870 (1.392–2.512)	

### Genotypes

The genotype distributions of *ADIPOQ* rs2241766, *UCP2* rs659366, and *FABP2* rs1799883 and their association with CRC risk are shown in Table 3. In the control group, the genotype distributions of the three SNPs were in HWE (χ^2^ = 0.290, P = 0.865; χ^2^ = 0.915, P = 0.633; and χ^2^ = 0.422, P = 0.810, respectively). With the TT genotype for *ADIPOQ* rs2241766 as reference, the OR for the TG genotype evidently increased (OR = 1.420, 95% CI 1.050–1.921), unlike for the GG genotype (OR = 1.384, 95% CI 0.782–2.451). Carriers of the TG+GG genotype for *ADIPOQ* rs2241766 increased the CRC risk, with an OR of 1.429 (95% CI 1.069–1.909). Compared with the GG genotype, the GA, AA and GA+AA genotypes for *UCP2* rs659366 were not associated with CRC risk (OR = 0.839, 95% CI 0.610–1.152; OR = 1.047, 95% CI 0.682–1.609; and OR = 0.888, 95% CI 0.658–1.197, respectively). The Ala/Thr, Thr/Thr, and Ala/Thr+Thr/Thr genotypes for *FABP2* rs1799883 did not increase CRC risk, with the Ala/Ala genotype as reference (OR = 0.893, 95% CI 0.656–1.216; OR = 0.909, 95% CI 0.526–1.570; and OR = 0.899, 95% CI 0.672–1.201, respectively).

**Table 3:** Association between *ADIPOQ* rs2241766, *UCP2* rs659366, *FABP2* rs1799883 and CRC risk.

Genotype		Cases N(%)	Controls N(%)	OR^a^ (95%CI)	P
rs2241766					
TT		173(43.2)	208(52.0)	Reference	0.064
TG		196(49.0)	165(41.2)	1.420(1.050–1.921)	0.023
GG		31(7.8)	27(6.8)	1.384(0.782–2.451)	0.265
TG+GG		227(56.8)	192(48.0)	1.429(1.069–1.909)	0.016
rs659366					
GG		147(36.8)	137(34.3)	Reference	0.423
GA		184(46.0)	202(50.5)	0.839(0.610–1.152)	0.277
AA		69(17.2)	61(15.2)	1.047(0.682–1.609)	0.833
GA+AA		253(63.2)	263(65.8)	0.888(0.658–1.197)	0.435
rs1799883					
Ala/Ala		230(57.5)	226(56.5)	Reference	0.759
Ala/Thr		139(34.8)	142(35.5)	0.893(0.656–1.216)	0.472
Thr/Thr		31(7.7)	32(8.0)	0.909(0.526–1.570)	0.731
Ala/Thr+Thr/Thr		170(42.5)	174(43.5)	0.899(0.672–1.201)	0.470

a Adjusted by confounding factors, including family per capita annual income, family history of CRC, sitting hours per day, BMI, smoking habit, alcohol-drinking habit and tea-drinking habit.

### Gene-gene and Gene-environment Interaction

ULR and GMDR were performed to explore the gene-gene and gene-environment interactions of *ADIPOQ* rs2241766, *UCP2* rs659366, *FABP2* rs1799883, and red meat consumption. As shown in table 4, by ULR analysis, the significant multiplicative gene-gene interaction between *ADIPOQ* rs2241766 and *FABP2* rs1799883 was detected, with an OR_multi_ of 2.013 (95%CI 1.131–3.646, P_multi_ = 0.017). The interactional indexes of additive model for *ADIPOQ* rs2241766 and *FABP2* rs1799883 were significant (RERI = 0.764, 95%CI 0.218∼1.311; AP = 0.514, 95%CI 0.165∼0.864; and S = −1.745, 95%CI is unachievable). Neither multiplicative interaction nor additive interaction between *ADIPOQ* rs2241766 and *UCP2* rs659366, or *UCP2* rs659366 and *FABP2* rs1799883 was significant. As shown in table 5, by GMDR analysis, the three-factor interaction model of *ADIPOQ* rs2241766, *UCP2* rs659366, and *FABP2* rs1799883 was the best model identified, with the minimum prediction error of 45.38%, the maximum CV consistency of 10/10, and a sign test P-value 0.0107. Collectively, a potential interaction of *ADIPOQ* rs2241766, *UCP2* rs659366, and *FABP2* rs1799883 on CRC risk may exist in our study.

**Table 4:** Gene-gene interaction for *ADIPOQ* rs2241766, *UCP2* rs659366, and *FABP2* rs1799883 on CRC risk.

Genotype	Genotype	Ca/Co^a^	OR^b^ (95%CI)	P	OR_multi_ b (95%CI)	P_multi_
rs2241766	rs659366					
TT	GA+AA	114/138	Reference		1.308(0.716–2.389)	0.382
TG+GG	GA+AA	139/125	1.287(0.900–1.841)	0.166		
TT	GG	59/70	0.975(0.629–1.511)	0.910		
TG+GG	GG	88/67	1.642(1.084–2.486)	0.019		
rs2241766	rs1799883					
TT	Ala/Thr+Thr/Thr	73/74	Reference		2.031(1.131–3.646)	0.017
TG+GG	Ala/Thr+Thr/Thr	97/100	0.959(0.617–1.489)	0.851		
TT	Ala/Ala	100/134	0.763(0.499–1.166)	0.211		
TG+GG	Ala/Ala	130/92	1.485(0.964–2.289)	0.073		
rs659366	rs1799883					
GA+AA	Ala/Thr+Thr/Thr	109/110	Reference		1.354(0.741–2.475)	0.324
GG	Ala/Thr+Thr/Thr	61/64	0.962(0.613–1.511)	0.867		
GA+AA	Ala/Ala	144/153	0.966(0.673–1.386)	0.851		
GG	Ala/Ala	86/73	1.259(0.826–1.919)	0.285		

a Cases and controls.

b Adjusted by confounding factors, including family per capita annual income, family history of CRC, sitting hours per day, BMI, smoking habit, alcohol-drinking habit and tea-drinking habit.

**Table 5:** GMDR models of high order interaction on CRC risk.

Model^a^	Prediction Error	P	Cross-validation Consistency
**Gene-gene interaction**			
rs2241766	0.4559	0.0547	10/10
rs2241766, rs1799883	0.4769	0.0547	8/10
rs2241766, rs1799883, rs659366	0.4538	0.0107	10/10
**Gene-environment interaction**			
Red meat	0.4141	0.0010	10/10
rs2241766, Red meat	0.4141	0.0010	10/10
rs2241766, rs659366, Red meat	0.4436	0.0107	6/10
rs2241766, rs1799883, rs659366, Red meat	0.4289	0.0107	10/10

a Adjusted by covariate, including family per capita annual income, family history of CRC, sitting hours per day, BMI, smoking habit, alcohol-drinking habit and tea-drinking habit.

The results of multiplicative gene-environment interactions on CRC risk are presented in Table 6. The interactional ORs for each of *ADIPOQ* rs2241766, *UCP2* rs659366, *FABP2* rs1799883 and red meat consumption were 1.222, 1.422, and 1.095 (all 95% CIs including 1 and all P_multi_>0.05), respectively. The gene-environment additive interactions were not detected, with the 95%CIs for RERI and AP including 0 and for S including 1 (data not shown). Table 5 shows the results obtained from GMDR analysis for one-factor to four-factor models adjusted by covariates mentioned above. The model of *ADIPOQ* rs2241766 and red meat consumption had the minimum prediction error of 41.41%, the maximum CV consistency of 10/10, and a sign test P-value 0.0010. The model including three SNPs and red meat consumption had the second minimum prediction error of 42.89%, the maximum CV consistency of 10/10, and a sign test P-value 0.0107, which indicated that potential interaction among the three SNPs and red meat consumption may exist in affecting CRC risk.

**Table 6:** Gene-environment interaction for each of *ADIPOQ* rs2241766, *UCP2* rs659366, *FABP2* rs1799883 and red meat consumption on CRC risk.

Genotype	red meat (times/week)	Ca/Co^a^	OR^b^ (95%CI)	P	OR_multi_ b (95%CI)	P_multi_
rs2241766						
TT	≤7	67/110	Reference		1.222(0.682–2.190)	0.500
TG+GG		77/104	1.251 (0.810–1.932)	0.312		
TT	>7	106/98	1.672 (1.100–2.543)	0.016		
TG+GG		150/88	2.558 (1.692–3.866)	<0.001		
rs659366						
GA+AA	≤7	91/133	Reference		1.422(0.773–2.616)	0.256
GG		53/81	0.967 (0.619–1.512)	0.884		
GA+AA	>7	162/130	1.661 (1.154–2.393)	0.006		
GG		94/56	2.286 (1.475–3.543)	<0.001		
rs1799883						
Ala/Thr+Thr/Thr	≤7	68/100	Reference		1.095(0.608–1.971)	0.763
Ala/Ala		76/114	0.973 (0.632–1.498)	0.900		
Ala/Thr+Thr/Thr	>7	102/74	1.773 (1.134–2.771)	0.012		
Ala/Ala		154/112	1.888 (1.261–2.827)	0.002		

a Cases and controls.

b Adjusted by confounding factors, including family per capita annual income, family history of CRC, sitting hours per day, BMI, smoking habit, alcohol-drinking habit and tea-drinking habit.

The cumulative effects of *ADIPOQ* rs2241766, *UCP2* rs659366, and *FABP2* rs1799883 combined with red meat consumption on CRC risk are shown in table 7. Based on the results in table 3, the genotypes of *ADIPOQ* rs2241766 TG+GG, *UCP2* rs659366 GG, and *FABP2* rs1799883 Ala/Ala were considered as risk genotypes. Subjects were divided into four subgroups by the number of risk genotypes. A significant dosage effect with an increasing number of risk genotypes was observed as the risk of CRC increased (P_trend_ = 0.037). Compared with subjects carried null risk genotypes, those carried three risk genotypes had higher CRC risk, with an OR of 2.243 (95%CI 1.196–4.207). Compared with subjects with low red meat consumption and null risk genotypes, those with high-red meat consumption and three risk genotypes had 3.439-fold CRC risk (95% CI 1.410–8.385). Plausibly, these results further provided the basis for the conclusion from GMDR that potential interactions among gene-gene and gene-red meat consumption may exist in affecting CRC risk.

**Table 7:** Cumulative effects of risk genotypes of *ADIPOQ* rs2241766, *UCP2* rs659366, and *FABP2* rs1799883 combined with red meat consumption on CRC risk.

	Total			Low-red meat (≤7 times/week)			High-red meat (>7 times/week)		
No.^a^	Ca/Co^b^	OR^c^ (95%CI)	P	Ca/Co^b^	OR^c^ (95%CI)	P	Ca/Co^b^	OR^c^ (95%CI)	P
0	46/50	Reference		20/26	Reference		26/24	1.194 (0.525–2.715)	0.672
1	158/172	0.920 (0.576–1.467)	0.725	59/92	0.743 (0.375–1.469)	0.392	99/80	1.321 (0.675–2.585)	0.417
2	142/151	0.992 (0.619–1.591)	0.973	48/81	0.704 (0.350–1.415)	0.325	94/70	1.514 (0.772–2.970)	0.228
3	54/27	2.243 (1.196–4.207)	0.012	17/15	1.571 (0.619–3.987)	0.342	37/12	3.439 (1.410–8.385)	0.007

a The number of risk genotypes which were defined as *ADIPOQ* rs2241766 TG+GG, *UCP2* rs659366 GG, and *FABP2* rs1799883 Ala/Ala.

b Cases and controls.

c Adjusted by confounding factors, including family per capita annual income, family history of CRC, sitting hours per day, BMI, smoking habit, alcohol-drinking habit and tea-drinking habit.

## Discussion

In this matched case-control study, ULR was used to assess the association of the IR-related gene polymorphisms and CRC, and GMDR and ULR were applied to explore the possible interactions of gene-gene and gene-red meat consumption on CRC.

IR has a strong genetic predisposition. Among the gene polymorphisms related to IR, *ADIPOQ* rs2241766, *UCP2* rs659366, and *FABP2* rs1799883 are the major hotspot loci. In this study, the TG+GG genotype of *ADIPOQ* rs2241766 increased CRC risk with an OR of 1.429 (95% CI 1.069–1.909) adjusted by confounding factors mentioned above, and may be an independent risk genetic variation for CRC. *ADIPOQ* located on chromosome 3q27 has been identified as a susceptibility locus for metabolic syndrome and T2DM, and is composed of three exons and two introns spanning a 17 kb region [34]. The *ADIPOQ* rs2241766 has been examined in several studies, and the results indicate that gene variation in rs2241766 is associated with decreased adiponectin level and increased IR [35], [36]. Adiponectin is an important adipocyte-derived plasma protein and is highly abundant in blood [37]. In contrast to other adipocytokines, adiponectin is significantly negatively associated with obesity, T2DM, and IR [38], [39], [40]. Adiponectin is also known as an insulin-sensitizing hormone, which can increase the effect of insulin on glucose metabolism. Thus, carriers of the *ADIPOQ* rs2241766 TG+GG genotype are more likely to be associated with lower adiponectin, higher IR and CRC risk compared with those carrying the wild TT genotype. Two recent studies in China [41] and Japan [42] indicated that a decreased level of adiponectin was a strong risk factor for both early and advanced CRC. The effect of adiponectin on CRC risk is biologically plausible [26]. It has been shown that high level of adiponectin inhibited the growth of malignant cells through stimulating adenosine monophosphate-activated protein kinase (AMPK) which might reduce the risk of developing cancers [43]. Adiponectin could suppress colonic epithelial cell proliferation via inhibiting the mammalian target of the rapamycin (mTOR) path way [44]. The anti-proliferative effect of adiponectin could also be partly explained by its selective sequestration of several mitogenic growth factors [45]. Another contribution of adiponectin to anti-carcinogenesis was promoting apoptosis via AdipoR1/APPL1 signaling and increased anti-oxidant potential [46]. What’s more, adiponectin may also work on anti-inflammation via inhibiting both the production of tumor necrosis factor-gamma (TNF-α) in macrophages and its action in endothelial cells [47], and inflammation on bowl was considered to be associated with CRC [48]. Researches on the relationship of *ADIPOQ* rs2241766 with CRC risk are limited and have varied conclusions. Most studies [49], [50], [51] have assumed that no relationship exists between *ADIPOQ* rs2241766 and CRC risk, whereas a study [52] made in Saudi Arabia concluded that the TG+GG genotype of *ADIPOQ* rs2241766 is a protective factor against CRC (OR = 0.41, 95% CI 0.19–0.86), which is inconsistant with our results. Further study must be conducted in a larger sample population to ensure the association of *ADIPOQ* rs2241766 and CRC risk.

Gene-gene interactions for *ADIPOQ* rs2241766, *UCP2* rs659366, and *FABP2* rs1799883 were estimated by ULR and GMDR. The results from ULR showed that both multiplicative and additive interactions for *ADIPOQ* rs2241766 and *FABP2* rs1799883 on CRC were significant, and the results from GMDR indicated that potential gene–gene interaction of the three SNPs seemed to predispose to CRC. FABP2 is an abundant cytosolic lipid-binding protein expressed exclusively in epithelial cells of the small intestine that is thought to participate in intracellular metabolism and the transport of long-chain fatty acids [53]. FABP2 influences the level of adiponectin may via the triglyceride. The variation of *FABP2* could affect the level of free fatty acid (FFA), and high FFA level can dose-dependently promote the triglyceride synthesis. With the increasing of triglyceride, the adipocyte secretion of adiponectin was inhibited, which resulted to the decreasing of adiponectin [54]. Furthermore, the growing synthesis of triglyceride stimulated the secretion of TNF-α which could negatively regulated adiponectin level [46]. Chamberlain et al. [55] found that with the variation of *FABP2* the human colonic carcinoma cell line cells increased the secretion of triglycerides, in vitro experiments. And they also found *FABP2* variation could increase the absorption and processing of fatty acids and then increase fat oxidation, which resulted to IR. A review performed by Weiss, et al. [56] in 2002 concluded that approximately half of the studies showed an association of the *FABP2* Thr54 allele with higher glucose tolerance or insulin action. Furthermore, in 3 of 4 studies in Japanese found an association of decreased insulin resistance with the *FABP2* Thr54 allele. Although no consistent conclusion was obtained, the interaction between *ADIPOQ* rs2241766 and *FABP2* rs1799883 on CRC was biologically plausible. *UCP2* was expressed in various tissues, including adipose tissue, and was placed in the inner mitochondrial membrane to regulate ATP synthesis [29]. Previous studies indicated that polyunsaturated fatty acids could stimulate *UCP2* expression through a peroxisome proliferation activated receptor-α (PPAR-α) mediated pathway, and TNF-α could increase mitochondrial oxidant production and induce expression of *UCP2*, which implied the potential interaction among UCP2, FABP2, and adiponectin. It has been demonstrated that UCP2 attenuates insulin secretion. By decreasing the coupling efficiency of oxidative phosphorylation, UCP2 lowers the ATP/ADP ratio, leading to the decreased stimulation of K_ATP_ channels and reduced insulin secretion. It may also work by decreasing reactive oxygen species (ROS) production, which is an important signal in glucose-sensing systems [57].*UCP2* is regarded as a candidate gene for obesity and T2DM. A study [58] performed among Spanish children and adolescents indicated that the *UCP2* rs659366 A allele potentially protected the obese population group against IR. D’Adamo et al. [59] performed a research demonstrating that the common rs659366 polymorphism in the *UCP2* contributed to IR by affecting insulin sensitivity. Thus, the interaction among *ADIPOQ* rs2241766, *UCP2* rs659366, and *FABP2* rs1799883 on CRC was biologically plausible.

The results from GMDR indicated that *ADIPOQ* rs2241766, *UCP2* rs659366, *FABP2* rs1799883 and red meat consumption potentially worked together in affecting CRC risk, and the results from ULR consistently supported the gene-environment interaction. A high-red meat diet results in high fat intake which can increase the plasma concentrations of triglyceride and insulin [60]. An experiment on mice indicated that a high-fat diet leads to elevated IR and associated enzyme expression in skeletal muscles [24]. Red meat is rich in iron which plays a direct and causal role in DM pathogenesis mediated both by β cell failure and IR [61]. Research has demonstrated that high red meat consumption is one of the convincing CRC risk factors [5], which is similar to the results of the present study that high-red meat consumption (>7 times/week) increased CRC risk. The mechanisms for the link between red meat and CRC may be attributed to high levels of fat associated with a high-red meat diet [62]. Heterocyclic amines (HCAs) found in meat cooked at high temperatures and polycyclic aromatic hydrocarbons (PAHs) found in meat cooked above a direct flame were both the cancerogen for CRC [63]. It can be concluded that red meat consumption may enhance the effect of the three SNPs on the risk of CRC.

The limitations in our study are listed as follows. First, we identified the lifestyle and dietary habits of the subjects in reference to 10 years before the disease diagnosis. Therefore, recall bias was difficult to avoid completely. We trained interviewers and recruited newly diagnosed CRC patients to reduce the bias. Second, the number of genes and SNPs related to IR in our study were limited, whereas many other genes were proven to be associated with IR. We must explore more genes and SNPs related to IR to assess accurately the susceptibility of genes for CRC.

In conclusion, this study represents the first report that IR-related gene polymorphisms exert their effects on CRC risk. The results showed that the gene polymorphism of *ADIPOQ* rs2241766 was associated with CRC risk. Furthermore, the interactions of *ADIPOQ* rs2241766, *UCP2* rs659366, *FABP2* rs1799883 and red meat consumption may contribute to the risk of CRC.
